# On the Unconscious Life of the Soul
*From Carus' *Psyche zur Entwickelungeschichte der Seele*.


**Published:** 1863-01

**Authors:** 


### Art. X.—ON THE UNCONSCIOUS LIFE OF THE SOUL.*

When we have brought clearly before our self-conscious consideration the extraordinary beauty and order with which, long before consciousness, the realization and sustentation of our bodily frames, as well as those of every living thing, are conducted, we are struck with a strange surprise. Nay, the further we dive into the depths of antecedent formation, the higher rises our veneration for this unseen power. Whoever has followed step by step, with persevering attention, the crystallizing process of even one single organism ; whoever has beheld how, through endless repetitions, the simple primary form of the microscopic germ within the ovum originates a peculiar cell formation, which is invariably the foundation from whence vessels, nerves, muscles, bones, with their peculiar radiations and metamorphoses, proceed; to such a one must it have gradually become apparent what wisdom, power, and beauty are required to make manifest, thus quite *unconsciously*, a self-individualizing Divine Ideal. And then the question naturally suggests itself: has the free operation of the *self-conscious* soul power to raise itself to a height of beauty, variety, and internal perfection equalling that which an unconscious operation of the psychical principle daily and hourly unfolds before our view ? After all that has been said respecting the relations and oppositions of nature and art, that art is never able in the smallest degree really *to create* has been invariably deduced ; and one must be entirely convinced that the inner perfection and highest physical adaptation of that *unconscious* power infinitely surpass everything that the *conscious* mind is similarly able to effect. Nay, when it becomes clear to us that everything we denominate *knowledge*† in the *conscious*

---

* From Carus' *Psyche zur Entwickelungeschichte der Seele.*

† I cannot forbear remarking on this point, how that the higher knowledge—that is to say, what we simply call knowledge, viz., the perception, the intuitive perception of the Ideal, itself rests entirely on the *unconscious* condition of many soul, is only a following up or searching after laws and relations, which are continually working themselves out *unconsciously* in the various existing realities in and around us, from the heavenly bodies down to the minutest blood corpuscle; then the Ideal universe rises before one as a proper circle, developing itself out of *unconsciousness* into *consciousness*, from whence it again descends to find its best satisfaction in the fullest comprehension of *unconsciousness* to which it can arrive: yet even then, that a complete attainment to nature through scientific constructions is an impossibility, and that something of a gulf must still ever remain between the two, is fully attested.

Even concerning man himself, we have opened before us remarkable evidences to this effect. When, for instance, we have convinced ourselves that the formation of this our organism, quite apart from all that conscious life shall subsequently develope in and from the same, exhibits a completeness, a variety, an inward suitability, which we meet with in no other organism, it must inspire us with peculiar admiration for mankind in general, quite irrespective of anything which man, as *conscious* individual, has irrespectively become; so that even in the individual man, who as yet, as a conscious intelligence, but insufficiently developes himself—nay, who often loses all dignity as a conscious being—we recognise none the less a wisdom, order, nay, a certain beauty in the internal life, which, the further we enter into the understanding of the phenomenon, must cause our wonder to increase.

By this veneration for unconsciousness, which, even without such scientific understanding, pervades, and has ever pervaded, mankind, much light is thrown on the imaginations of men, even in the earliest ages of the world: the peculiar reverence for the nature of children, even before any higher *self-conscious* life had developed itself in them, the horror of manslaughter amongst the Hindoos, and amongst so many nations the worship of the human image, as well as of many animal forms, as divine. Undoubtedly, the more backward they were in knowledge the more errors found their way into their conceptions on this point; so that what was only *divine*, a single ray of that which, by us, is considered the one and only absolute God, was often received as Godhead itself; and precisely in this manner was the error of Pantheism originated. This Pantheism, the opinion that an absolute Godhead could be com-

---

previously existing ideas. As Deucalion, according to the myth, threw stones behind him, out of which proceeded men—so in like manner, the soul casts behind it, into *unconsciousness*, multitudes of childish thoughts, from whence (such is the condition comprehended) immortal ideas subsequently arise.

prised in every separate particle, stands in direct opposition to what one may most properly call *Entheism:* that is to say, the recognition of *the Divine in everything.* And as certain as it is that this Entheism indicates the only sound proper view of the universe, so is it also sure that a true, direct pantheism, like real atheism, is too great an absurdity ever to have satisfied man in any developed stage of intelligence which can be properly so called.

It has been already said, in the opening chapter of this work, that it was very difficult to grasp any true idea of the *unconscious* existence and working of the soul in the sphere of *consciousness,* yet at the same time that it was *there only* that *any key to true psychology could be found.* Let us next endeavour to consider attentively how much there is even in the soul's *conscious* condition which works and fulfils itself quite *unconsciously.* It is undoubtedly proved, for instance, that the muscles which produce the action of the lungs obey the voluntary impulse of our conscious psychical life through the operating upon them of nervous action. We can for a while check this motion, accelerate, interrupt, strengthen, or weaken it intentionally, thus proving its complete dependence on our *consciousness.*

None the less does this action in its *regular* and *permanent* course proceed throughout our whole life, for the most part *unconsciously;* thus evidencing that there is a very moveable boundary between the *conscious* and the *unconscious,* and that both are rays from one and the same source. This is perhaps rendered still more conspicuous in any actions that have the least connexion with art. Here, quite in the sphere of *consciousness,* and irrespective of any arbitrary subjection of the muscles, is what we call *execution;* that is, nothing else but an exertion which brings back into the sphere of *unconsciousness* something which appertains to *consciousness.* Pianoforte playing, for instance, each particular touch, each note the finger strikes, is originally voluntary, and must be produced at first by particular, intentional, and voluntary nervous action on the proper muscles. In whatever manifold quantity they are brought forth, however often repeated, they pass away by degrees in their several complications into *unconsciousness,* and are so far withdrawn from *consciousness* that they are never considered to be again singly required; but the idea of realizing, in a general manner, certain melodies is quite sufficient for them to be produced in their proper time and connexion, as *unconsciously* as the motions of the lungs follow each other without any *conscious* consideration.

The same is the case with the exercise of our most essential movement, *walking*, and with a hundred others, from which it is clearly proved that in the power of *doing*, as well as in *knowing*, the passing out of *consciousness* into *unconsciousness* comprises, properly speaking, the summit of human completeness. The latter is a consideration which deserves the full attention of psychologists, and which has never, properly speaking, been pursued, though E. Stahl has bestowed much particular attention on a great deal of the same sort. It is certainly very remarkable that for the powers of *doing* and *performing*, human *art* has traced out the same paths as *science* has for those of knowledge and perception. For, as the higher attainments in science are supposed, the further the *conscious* intellect of man is able to penetrate to the true perception of laws and ideas which *unconsciously* work themselves out in our own organism, and in that of the world-phenomena around us; and as the chief proposition in the doctrines of psychology is to penetrate the regions where psychological life as yet quite *unconsciously* reveals itself; so also will each power of action only really become art, which accomplishes everything, in so far as it follows the dictates of a conscious purpose of the will, and is on the other side in and for itself *unconsciously* fulfilled. And the production being brought into the highest state of preparation possible for the facilitation of the action, it becomes now indispensable that the soul should realize sensibly and particularly all the special purposes of the will, which are necessary to bring any intended design into execution; where now, *with the purpose to attain it*, there remains but that the design should float clearly and distinctly before the mind's view, for the active skill boldly and easily to work out its accomplishment.

If we turn, moreover, to that which in *conscious* life we call knowledge or perception, we shall presently understand, when we consider the procession of these from *unconsciousness*, why Plato represented all acquisition of knowledge as a *remembrance* (*Erinnern*)—"to find inside"—to find already there, where hitherto was no knowledge *whatever*, but where at the same time this reality, this ideal, was still existing like the unconscious embryo in the conscious mother; on which account Socrates has so often compared the development of thought, that is to say, the attainment to higher knowledge, to an act of midwifery. All this, then, points with certainty to the rich and wonderful world which we carry darkly within us, and every consideration of this sort must enable us to form a still clearer idea of the relations between *consciousness* and *unconsciousness*. A still brighter light may be thrown upon it if we consider the very gradual appearance of particular innate tendencies of the conscious soul.

Then it is instantly seen how far we must go back into the history of our ideal being, and at the same time into the sphere of our unconscious existence, if we wish to attain to any discovery as to the first commencement or peculiarities of this existence. I would begin here by recalling how many special properties of conscious psychical life, how many peculiar tendencies of the mind, how many artistic talents, become in this manner the property of people, for which, though they may not be developed for some time to come, the capacity must have existed in them from the first. Now we must be quite aware in what a perfectly *unconscious* condition the soul finds itself at that first period of formation within the ovum, when none of these things could possibly exist; we see clearly how here, in the soul of the embryo, whilst realizing itself so fully as a developing and formative power, drawing forth and apportioning the material, are still *unconsciously* prefigured all the self-informing mental properties of *conscious* life; and we have thus before us the most remarkable and instructive insight as to the nature of the relations between *unconsciousness* and *consciousness*. Undoubtedly, it must thus become obvious how thoroughly our *conscious* life rests on and proceeds from the sphere of the *unconscious:* how, properly speaking, it is the first creative act of the pre-existing *ideal*, to consolidate quite *unconsciously* the wonderful variety of the organism, and that when, through the reflection of the ideal, *consciousness* has insinuated itself into this creation, the *unconscious* radiation of this divine idea is the perennial fountain from whence are ever proceeding fresh treasures by which consciousness is enriched.

---

